# Advancements in Ambient Ionisation Mass Spectrometry in 2024: An Annual Review

**DOI:** 10.1002/ansa.70007

**Published:** 2025-03-22

**Authors:** Alisha Henderson, Liam M. Heaney, Stephanie Rankin‐Turner

**Affiliations:** ^1^ School of Sport, Exercise and Health Sciences Loughborough University Loughborough UK; ^2^ Department of Chemistry University of Pittsburgh Pittsburgh Pennsylvania USA

## Abstract

The introduction of ambient ionisation mass spectrometry (AIMS) is among the most important developments in analytical chemistry in recent years. Enabling the direct analysis of samples in their native state with neither sample preparation nor chromatographic separation provides chemical characterisation in a matter of seconds. Since its inception in the early 2000s, the field of ambient ionisation has continued to expand and develop, pushing the boundaries of analytical chemistry and finding utility across broad areas of scientific research. This annual review provides an overview of key developments and applications of AIMS throughout 2024. The introduction of novel ion sources, development of existing techniques for enhanced performance, and diverse applications across forensic science, food chemistry, disease detection and more highlight the expanding versatility and widespread adoption of ambient ionisation across the globe.

## 1 Introduction

Mass spectrometry has been at the forefront of analytical chemistry for decades, providing a powerful tool for the sensitive and quantitative detection and identification of diverse analytes. In the early 2000s, the face of mass spectrometry drastically changed with the introduction of ambient ionisation mass spectrometry (AIMS). The first AIMS techniques, desorption electrospray ionisation (DESI) [1] and direct analysis in real‐time (DART) [2], suddenly provided researchers with the ability to perform real‐time analysis of samples in their native state, forgoing the lengthy and costly sample preparation and chromatography steps limiting traditional techniques. As we pass the 20th anniversary of the introduction of ambient ionisation, we have inevitably witnessed this powerful suite of techniques rapidly adopted across diverse areas of research, ranging from clinical diagnostics and pharmaceutical research to forensic investigations and environmental monitoring. Each year, AIMS take steps towards more widespread adoption. Ion sources are modified to enhance performance and reduce inherent weaknesses, techniques are applied in new and exciting areas of research, entirely novel ion sources are introduced, and portability is explored to take mass spectrometry out of the laboratory and into the world.

This review provides an overview of developments and applications in ambient ionisation throughout 2024, building upon our prior annual reviews [3, 4, 5]. As the adoption of AIMS expands beyond what can be covered in a single review, this work focuses on major developments in the field and key applications using AIMS with no or minimal sample preparation, excluding studies that incorporate extensive sample preparation prior to analysis. Search terms included ‘ambient ionisation’ and individual AIMS technique names using the PubMed database in October and November of 2024. A brief overview of the techniques covered in this review is detailed in subsequent sections, followed by detailed coverage of this year's developments and applications in ambient ionisation.

## 2 AIMS Techniques

Since its introduction over 20 years ago, the field of AIMS has exploded, resulting in the development of dozens of diverse ion sources fit for a variety of sample types and target analytes. The techniques available broadly fall into three primary categories depending on the sampling and ionisation mechanisms of each ion source. Liquid extraction techniques involve direct extraction or desorption of analytes from the surface of the sample, incorporating an ionisation process based on electrospray ionisation (ESI). Plasma‐based techniques utilise an ionising plasma to which samples are directly exposed, inducing the near‐instantaneous desorption of analytes and ionisation by mechanisms most akin to those in atmospheric pressure chemical ionisation (APCI). Laser ablation techniques use infrared or ultraviolet lasers to achieve the ablation and desorption of analytes from a sample surface, some incorporating diverse ion sources to support ionisation following ablation.

Plasma‐based AIMS techniques account for a quarter of publications using ambient ionisation in 2024, with DART at the forefront. DART uses an inert gas exposed to an electrical potential to induce the production of excited‐state species, which act as reagent ions [2]. These reagent ions are introduced to the sample positioned in between the ion source and the MS inlet, inducing the desorption and ionisation of analytes either directly through contact with the metastable species or indirectly through reactions with ions derived from the atmosphere. Another commercially available and user‐friendly ion source is the atmospheric solids analysis probe (ASAP) [6]. This technique uses a glass sampling tool to collect a small amount of liquid or solid sample, which is then inserted into the instrument to directly expose the sample to the ion source. A stream of heated gas thermally desorbs analytes from the tip of the probe, followed by APCI. ASAP has gained particular popularity due to its ease of use, being readily utilised by workers and students with minimal training [7, 8]. Finally, many plasma‐based ion sources can be very simply constructed, making them a popular choice for those without access to specialised or commercial instrumentation. Dielectric‐barrier discharge (DBD) incorporates two electrodes separated by an insulating barrier, such as glass [9]. Application of a high‐voltage alternative current between the electrodes causes the formation of a non‐thermal plasma into which gas‐phase or vaporised analytes can be introduced. Several other sources operate under similar principles but with slightly modified geometries and mechanisms, such as LTP and hollow cathode discharge (HCD), some of which extend the plasma outside of the source for direct application to solid and liquid matrices. DBD has since been developed into a commercial ion source known as SICRIT, or soft ionisation by chemical reaction in transfer, joining the growing list of AIMS techniques to become commercially available.

As one of the original AIMS techniques introduced by the Cooks group in the early 2000s, DESI is amongst the most widely used and best developed [1]. DESI uses a stream of charged microdroplets, which are directed at the surface of the sample. As charged droplets collide with the sample, secondary droplets are produced, propelling the desorbed and ionised analytes towards the MS inlet. The success of DESI has sparked the development of various derivatives, such as air‐flow‐assisted desorption electrospray ionisation, or AFADESI, which supplements ion desolvation and transportation using a high‐flow air stream [10]. Another adjacent technique is nano‐DESI, which achieves analyte desorption from the sample surface via a self‐aspirating nanospray that forms a liquid bridge between two capillaries, ultimately directing desorbed ions into the inlet [11]. In all, DESI and its derivatives account for 22% of publications covered in this review, making it one of the most popular AIMS techniques available, particularly finding its niche in tissue imaging. Shortly after the introduction of DESI, the same group developed paper spray ionisation (PSI) [12]. This ESI‐based technique consists of a triangular paper substrate onto which a sample is applied, followed by the application of a spray solvent and high voltage. This combination induces the electrospray of analytes from the tip of the paper positioned in front of the MS inlet, with the paper itself reducing the undesirable effects of interfering species in complex matrices. PSI has since formed the basis of several other techniques, including cone spray ionisation, which is essentially a three‐dimensional paper spray source, enabling solid samples to be analysed directly [13].

Probe electrospray ionisation (PESI) was developed by the Hiraoka group in 2007 [14]. PESI briefly touches a solid needle to the surface of a sample, collecting a small amount of material, which is electrospray from the needle tip upon application of a high voltage. The technique was subsequently modified into sheath‐flow PESI (sfPESI), which encloses the needle in a solvent‐filled capillary in order to make PESI additionally applicable to solid samples via liquid extraction from the surface [15]. The MasSpec Pen similarly utilises liquid–solid extraction for the direct sampling of materials [16]. This handheld tool directs solvent to the surface of the sample to extract analytes, after which the droplet is transported through a sampling line to the mass spectrometer for ionisation. Ionisation was traditionally achieved using inlet ionisation, though the MasSpec Pen has been coupled with alternative ion sources such as APCI. Extractive electrospray ionisation (EESI) incorporates two sprayers, one introducing a charged spray solvent and the other a nebulised sample [17]. The two sprayers intersect in front of the mass spectrometer inlet, resulting in the ionisation of analytes before sampling into the MS inlet. Secondary electrospray ionisation, or SESI, follows a similar process, though it introduces samples in the gas phase rather than as a nebulised liquid sample, making it readily applicable to gaseous samples such as exhaled breath [18].

Although less commonly used than their liquid extraction and plasma‐based counterparts, laser ablation AIMS techniques provide a means for highly focused analyte desorption and ionisation that is particularly ideal for imaging. Amongst the most commonly utilised is laser ablation electrospray ionisation (LAESI), which harnesses an infrared laser to ablate analytes from the sample surface, followed by ionisation using an adjacent ESI source [19]. Whilst ESI is most commonly used for ionisation in this case, other ion sources have been coupled with laser ablation, including DBD. Finally, some ambient ion sources involve alternative or combined desorption and ionisation processes that are not described by the three aforementioned categories. One such technique is rapid evaporative ionisation mass spectrometry (REIMS), which induces sample vaporisation and ionisation using an electrocautery mechanism [20]. More commonly referred to as the iKnife, this handheld device can be readily applied to a range of sample types, but it is most typically utilised in the analysis of biological tissue samples.

**FIGURE 1:**
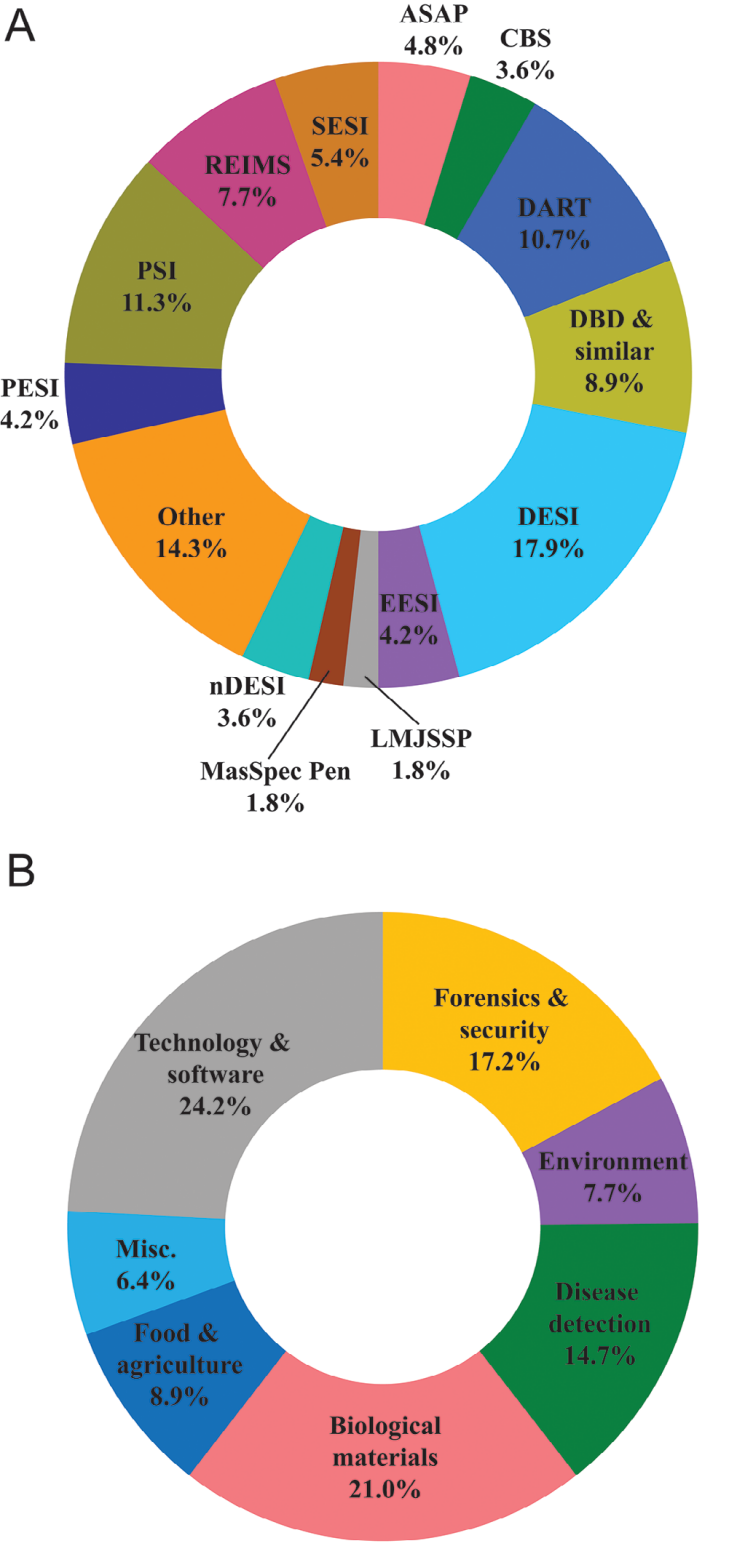
Distribution of papers detailed in this review using each ambient ionisation mass spectrometry technique (A) and the relative contribution of papers to each field of research (B). See Table 1 for technique abbreviations. Techniques with less than three publications are combined in the ‘other’ category of A.

Whilst the techniques described here are by no means inclusive of the dozens of ambient ion sources developed over the past 20 years, they account for the vast majority of studies covered in this review. AIMS techniques covered in this review are summarised in Table 1 and further represented in Figure 1. The subsequent sections explore the diverse scenarios in which these techniques have been applied and highlight recent technological and methodological developments throughout 2024.

**TABLE 1:** Summary of techniques covered throughout this review.

Technique	Abbreviation	Classification
Air‐flow‐assisted desorption electrospray ionisation	AFADESI	Liquid extraction
Atmospheric pressure solids analysis probe	ASAP	Plasma
Coated blade spray	CBS	Liquid extraction
Cone spray ionisation	CSI	Liquid extraction
Crystallisation and solvent evaporation ionisation	CSEI	Liquid extraction
Direct analysis in real time	DART	Plasma
Dielectric‐barrier discharge	DBD	Plasma
Desorption electrospray ionisation	DESI	Liquid extraction
Desorption, separation and ionisation	DSI	Plasma
Extractive electrospray ionisation	EESI	Liquid extraction
Internal extractive electrospray ionisation	iEESI	Liquid extraction
Laser ablation electrospray ionisation	LAESI	Laser ablation
Liquid micro junction‐surface sampling probe	LMJ‐SSP	Liquid extraction
Low‐temperature plasma	LTP	Plasma
Magnetic particle spray	MPS	Liquid extraction
MasSpec Pen	—	Liquid extraction
Nanospray desorption electrospray ionisation	Nano‐DESI	Liquid extraction
Probe electrospray ionisation	PESI	Liquid extraction
Paper spray ionisation	PSI	Liquid extraction
Rapid evaporative ionisation mass spectrometry	REIMS	Other
Secondary electrospray ionisation	SESI	Liquid extraction
Sheath‐flow probe electrospray ionisation	sfPESI	Liquid extraction

## 3 Applications and Developments

### 3.1 Food and Agriculture

The need for analytical testing in the food and agriculture industries broadly spans quality control to ensure desired flavours, the detection of contaminants that may be harmful to consumers, and the identification of adulterated and counterfeit products. The sheer scale of products requiring testing in the food industry, and the tight timeline on which it must be achieved, make AIMS particularly attractive for real‐time monitoring in this industry.

Illegal adulteration of food and origin fraud are common practices worldwide and often challenging to detect, especially given the scale of food products requiring screening. The verification of authentic food products using ambient ionisation, particularly in combination with multivariate statistical analysis to create predictive models, has exhibited notable attention in the past year. ASAP‐MS has been reported in the rapid detection of adulterated cinnamon oils [21], whilst coated blade spray (CBS) and DBD have both been used for the rapid authentication of honey samples to determine botanical origin [22, 23]. Leite et al. used PSI to create metabolic profiles for garlic, the flavour profile of which can drastically differ depending on the geographical region [24]. Analysing samples from Brazil and China, the combined PSI and multivariate analysis enabled clear differentiation between samples, with Brazilian garlic exhibiting higher levels of amino acids and organosulfur compounds. In similar efforts to understand the effect of geographical origin on wine, another study used µPESI for the rapid chemical profiling of red wine from Australia, Chile and China [25]. Based on PESI, µPESI uses a fine stainless steel needle with a ditch laser‐etched into the tip, intended to improve precision. In this work, red wines from different geographical regions were readily discriminated with the use of PCA, in particular, finding amino acid profiles distinctive to each region. Though µPESI was able to readily differentiate wines from different regions, the improved precision proposed by needle etching only offered %RSD values that were similar to those previously seen with other ambient ionisation methods, including PESI [26]. Furthermore, the analysis time of 1.2 min per sample is, while rapid in comparison to traditional LC/MS and GC/MS methods, somewhat longer than many other AIMS approaches. Aside from geographical authentication, AIMS has been readily utilised to detect and prevent food fraud. In recent work, PSI was used to analyse of the unsaponifiable fraction of olive oil, which is of particular interest as a health supplement and is susceptible to food fraud [27]. Oil samples were applied directly to the PSI substrate, though drying the paper before the application of a high voltage was found to be critical for optimum signal intensity. Ultimately, three key categories of olive oil could be readily differentiated with a 95% predictive accuracy. Finally, whilst a broad range of AIMS techniques have been used for food authentication purposes, REIMS consistently emerges as a popular technique of choice. In 2024, it was broadly used for the authentication of beef [28, 29], fish [30] and plant oils [31], highlighting its versatility and usability in the context of food authentication.

Whilst freezing food is a common approach to preserving the quality of the product, repeated freezing and thawing can result in a contrary effect, decreasing the quality for consumption. As such, the ability to detect whether a product has been subject to repeated freeze‐thaw cycles can provide information on both food quality and a product's storage history. Shen et al. investigated the use of REIMS for the real‐time determination of tuna freeze‐thawing [32]. Frozen tuna samples were grouped according to the number of freeze‐thaw cycles they had endured and compared to fresh tuna. Through lipidomic profiling, they found the fatty acid profile of the fish to be a good marker of storage conditions, revealing 13 fatty acids to be significantly different between fresh tuna samples and those that had been through multiple freeze‐thaw cycles. There was no significant effect between the fresh tuna group and the single freeze‐thaw group. The same group shared similar findings using REIMS in a simulated cold chain interruption study of salmon, observing an increase in fatty acid abundance in salmon samples that had been through multiple freeze‐thaw cycles [33]. The study performed a more in‐depth investigation into the process of lipid oxidation and its effect on food quality, including flavour and colour. The authors concluded that there was a positive correlation between lipid oxidation and the number of freeze‐thaw cycles, which were rapidly detectable by REIMS. Given the complexity and heterogeneity in food products, a key challenge when sampling food surfaces directly is the optimisation of ion source geometries to ensure maximum desorption and ionisation efficiency. Bai et al. modified a DART ion source through the development of an angled sampling probe, altering various source parameters, including a reduced source outlet aperture and incorporating a curved sample inlet [34]. When these optimised parameters were used to sample fruit surfaces with a range of textures and shapes to evaluate the performance. The modified DART source was able to sample the different fruit surfaces directly with no sample pretreatment. For example, the successful sampling of cherry radishes, which have a higher water content, highlights the versatility of the technique to directly sample different texture surfaces and sample types. However, the authors noted that a key limitation of the technique is the comparatively low spatial resolution for imaging compared to more widespread imaging techniques such as DESI or MALDI.

In addition to food quality, the rapid analytical capabilities of AIMS are well‐suited to ensuring food safety by providing fast results to prevent the consumption of unsafe products. DART‐MS, ASAP‐MS and ion mobility spectrometry (IMS) were used as part of a rapid screening method to detect opioids in poppy seed samples to maintain food safety, driven by the hundreds of incidents of consumption of contaminated poppy‐based products in the United States [35]. Sixteen poppy seed samples were analysed using the three techniques alongside a negative control to evaluate their ability to determine the presence or absence of opioids in food samples. The DART‐MS method exhibited the highest rate of true and partial positive results at 87%, whilst the ASAP‐MS showed a higher rate of false negatives (31%). In addition to the performance of the screening process by the instruments, the application and potential portability of the systems were also considered, with AIMS systems holding great potential to be incorporated into large‐scale point‐of‐need screening protocols for food safety. Despite having the highest true positive percentage, the DART‐MS system used was the least portable, requiring a larger number of components for analysis. The balance between performance, usability and portability needs to be considered in future work when discussing the potential for in‐field analyses. There is also significant interest in understanding the physiological effects of exposure to unsafe food products. A recent study used LA‐REIMS to evaluate metabolic changes in pregnant women who had been exposed to aflatoxins via food, a toxic fungal metabolite that is a significant worldwide threat to food safety [36]. The LA‐REIMS technique was used for the rapid profiling of untreated serum collected from pregnant women in a rural region of Ethiopia. Through the comparison of almost 2000 metabolic features across non‐exposed and exposed groups, it was determined over 81% of the women exhibited signs of exposure to aflatoxins. This study not only identified potential markers of aflatoxin exposure but highlighted the potential to employ rapid techniques such as REIMS on a larger scale to help identify communities at higher risk of exposure. A separate publication by Cai et al. developed a screening method using DESI‐MS for the rapid detection of shellfish toxins in urine samples [37]. With a particular focus on paralytic shellfish poisoning, the most common type of shellfish poisoning, they demonstrated the ability to use DESI as a rapid urine screening tool to detect key markers of a range of different toxins. The DESI approach performed comparatively well against a more standard LC/MS method, achieving similar inter‐ and intraday precision. However, the LOD for DESI was notably poorer, in the range of 87–265 µg/L in comparison to 2.2–14.9 µg/L by LC/MS, the sensitivity using AIMS still largely met requirements for rapid screening of shellfish poisoning.

### 3.2 Forensics and Security

Forensic analysis relies heavily on rapid and accurate results to ensure the timely progression of criminal investigations. The traditional mass spectrometric techniques utilised in forensic analysis, namely GC/MS and LC/MS, are well‐established and robust, long since regarded as the gold standards for the analysis of drugs, explosives and toxins. However, the requirement for transportation of materials to laboratories, large evidence backlogs, destructive sample preparation, and, at times, lengthy chromatographic run times all result in hindered evidence processing times. This bottleneck could be eased by the development of more rapid analytical tools to allow real‐time screening of suspected materials, either in the laboratory or on‐site at crime scenes with portable instrumentation. With this in mind, ambient ionisation has naturally garnered extensive interest within the forensics community.

AIMS has been a key tool in the rapid analysis of explosives. Forbes et al. developed an in‐line thermal desorption system coupled with a DBD ion source for the analysis of explosives, propellants and post‐blast debris [38]. Sample wipes were used to collect materials of interest before being thermally desorbed and vapours directed into the DBD ion source. Sub‐nanogram detection limits were achieved for a range of common explosives, including TNT, RDX, PETN and HMX. Bain et al. had a similar goal, this time using 3D‐printed cone spray ionisation for the rapid analysis of post‐blast and post‐burn materials [39]. This particularly convenient technique enables untreated solid material to be added to the hollow cone for direct analysis in a process similar to PSI. Estevanes et al. took a somewhat similar approach in developing a simple filter paper sampling method for post‐blast debris followed by rapid analysis using DART by direct exposure of the filter paper to the ion source [40]. The study further evaluated the filter paper collection method with different surfaces, finding that the surface from which explosive material was sampled had a notable effect on the ability to positively identify analytes. The same group also compared DART with Raman microscopy in the analysis of various explosives, demonstrating the advantages of DART for sensitivity but highlighting the benefits of combining the technique with Raman microscopy, which was demonstrated to be more suited to the analysis of inorganic oxidising salts [41]. A dual HCD ion source was furthermore introduced for the ionisation of vapours of explosives [42]. The source incorporated two separate sampling inlets for both positive and negative ion production, enabling the formation of multiple ions to support analyte identification.

The simple and user‐friendly nature of certain AIMS techniques, in combination with their potential for field‐portability, has drawn particular interest in the analysis of illicit substances. The potential for police forces to perform rapid on‐site screening of suspected drugs could significantly reduce laboratory workloads and, in turn, analytical wait times. Since its commercialisation, the use of ASAP as a forensic analysis tool has continued to expand, especially in rapid drug analysis. This has particularly been applied in the context of real‐world samples, being utilised to screen ecstasy tablets seized by police forces in Brazil [43] and on‐site with portable instrumentation at UK music festivals to verify the content of drugs sourced from amnesty bins [44]. While not yet optimal for quantification, ASAP has clear utility in the rapid screening of drugs to provide a real‐time assessment of potential illicit substances and hazardous additives, particularly crucial with growing concerns surrounding the adulteration of drugs with lethal substances such as fentanyl. Efforts have been made to improve the quantitative capabilities of ASAP, with recent work exhibiting good analytical performance in drug analysis through the incorporation of internal standards [45]. In similar efforts to evaluate AIMS in a real‐world context, the Mulligan group worked with local law enforcement agencies to compare spray‐based ambient ionisation techniques coupled with a portable mass spectrometer for the screening of cannabinoids seized during legal operations [46]. The study systematically compared paper spray and filter cone spray ionisation and different solvent systems, ultimately showing that the ion source and its parameters dramatically affect the ability to detect and confirm the identity of cannabinoids. The study highlights that, although AIMS with portable technology holds great potential for use by police forces, extensive research is needed before such tools can be rolled out and used by non‐experts. Other studies have seen PESI applied to the detection and quantification of 89 benzodiazepines, demonstrating excellent agreement with LC–MS/MS [47], DART used to detect illicit substances from drug‐laced weigh paper from police casework [48], and a combination of surface‐enhanced Raman spectroscopy with PSI for fentanyl analysis, in which a dual purpose paper substrate was used for both Raman and PSI [49].

AIMS is not only a powerful tool for determining the identity of illicit drugs, but the past year has seen a marked increase in the use of AIMS to detect forensically relevant drugs and toxins in biological materials, both in mocked spiked samples and casework samples. The Manicke group used PSI‐MS with integrated solid‐phase extraction for the detection of drugs in plasma [50]. The online SPE stage extracts and concentrates analytes from the plasma immediately prior to PSI. Over 300 authentic samples from overdose cases were screened for 160 different drugs, including cannabinoids and fentanyl analogues, achieving sub‐nanogram detection limits. With a similar goal, fibre spray ionisation using polymeric capillary fibres was demonstrated to be able to detect cocaine and its metabolites in blood samples from suspected drug users, proving to be comparable to the more established PSI [51]. DART has also been explored as a tool to detect drugs in biofluids. A recent study used DART to detect new psychoactive substances in urine, with samples introduced into the ion source via paper sample holders. The authors were able to detect 21 drugs of interest, utilising MRM mode to boost selectivity without the need for chromatographic separation and achieving 70%–100% agreement with a more standard LC–MS/MS method [52]. Zhang et al. developed low‐temperature arc plasma ionisation and, in combination with Leidenfrost‐assisted thermal desorption, were able to detect furanyl fentanyl in urine, beverages, and lake water [53]. Low pg/mL detection limits were achieved despite the complex nature of some of the matrices. Li et al. combined magnetic dispersive solid‐phase extraction with DART‐MS for the rapid quantification of 21 synthetic cathinones in urine samples [54]. All target analytes could be detected in <1 min down to 0.5 ng/mL in good agreement with a standard LC–MS/MS method. Finally, a rapid PESI method, termed RaDPi‐U, was developed by the Zaitsu group, achieving the high‐throughput detection of 40 drugs in human urine [26]. The technique was applied to both spiked urine and postmortem samples, demonstrating its applicability to real casework samples. PESI has generally gained popularity as a key technique for detecting controlled substances in biofluids, with other studies showing its use to detect glyphosate and glufosinate in human blood, herbicides commonly used in intentional poisonings and suicide cases [55], and cocaine and its metabolites in saliva [56].

PESI has more generally found increasing use in the field of forensics and security, most recently in the analysis of drugs in fingerprints. Kim et al. demonstrated the ability of sfPESI to detect drug residues in fingerprints and forensic tape lifts, demonstrating a fascinating new application of the technique and expanding the forensically relevant information that could be gleaned from a fingerprint [57]. In addition to this work, two other studies utilised DESI to image fingerprints, one through the detection of the fingerprint enhancer oil Red O [58] and the other visually imaging gelatin‐lifted prints and detecting both endogenous and exogenous metabolites [59]. While forensic applications of AIMS are largely centred around drugs and explosives, other areas of interest in forensics have been explored. The liquid microjunction surface sampling probe (LMJ‐SSP) was applied to the analysis of lubricants, spermicides and other condom residues in an effort to demonstrate the ability to detect prophylactic use in sexual assault cases where DNA evidence is not obtained [60]. The authors were able to detect the spermicide nonoxynol‐9 on a variety of surfaces and differentiate between different condom brands and types. Mörén et al. developed a thermal desorption DART method for the rapid analysis of a panel of multiple riot control agents across 20 commercial self‐defence sprays [61]. Samples were applied to a specially designed glass fibre swab or cotton fabric prior to thermal desorption and ionisation, demonstrating the ability to identify target analytes in both pure solutions and commercial products, furthermore detecting analytes up to 3 weeks after application of the sprays. Finally, DART has also been applied in efforts to detect illegal trade. In a recent interlaboratory cross‐platform study, DART was shown to be robust in the identification of wood species, supporting efforts to tackle illegal logging and trade through the development of a mass spectral database of different tree species [62]. The growing evaluation of AIMS techniques across multiple laboratories will be crucial in supporting the widespread adoption of techniques such as DART for forensic purposes, with there currently being limited data on interlaboratory protocols and results.

### 3.3 Disease Detection

Early diagnosis of infection and disease is crucial for a positive patient outcome, both to identify the disease before it progresses and to establish appropriate treatment plans. Current diagnostic strategies frequently utilise laboratory analyses of biological specimens to measure key biomarkers, guiding subsequent testing and treatment. In many regions of the world, wait times associated with such analyses are long, resulting in the progression of disease and, in turn, increased avoidable fatalities. The development of analytical tools to screen for and detect disease‐associated biomarkers has the potential to significantly advance disease diagnostics, resulting in international efforts to drive forward ambient ionisation in this field.

With cancer being amongst the leading causes of death each year, cancer diagnostics have been a particularly popular focus area for AIMS research. In a recent publication by Zheng et al., internal extractive electrospray ionisation mass spectrometry (iEESI‐MS) was used to create a diagnostic model for colorectal cancer through untargeted metabolic profiling [63]. Metabolic profiles of cancerous and healthy tissues were acquired, followed by the use of machine learning to create a diagnostic model, ultimately achieving a prediction accuracy of 88%. Gene expression profiles were then linked to disease‐associated metabolic changes to better understand the link between metabolic disorders and the genome. Manaprasertsak et al. applied DESI‐MS to explore the metabolic effects of cancer on human tissues [64]. A known metabolic change during the growth of cancer cells is the increased uptake and synthesis of cholesterol. With this in mind, they hypothesised that this effect would result in alterations to the cholesterol molecule and detectable variation in cholesterol fragmentation and isotope patterns. To investigate this hypothesis, they used DESI‐MS and time‐of‐flight secondary ion mass spectrometry to map the distribution of cholesterol in mice that develop breast cancer. Intact and fragmented cholesterol were measured in both cancerous and healthy tissues, demonstrating the abundance of fragmented cholesterol was significantly higher in the cancerous tissues compared to the control healthy tissues. This was confirmed using DESI as an alternative ionisation technique, though there was some variation between the two methods. They concluded that the observed increase in fragmented cholesterol in cancerous tissues could indicate a change in the molecular integrity of cholesterol in cancerous cells, and future work could use this approach to monitor these changes through tumour progression. Numerous other studies have employed AIMS for the study of cancerous tissues, including DESI to study the mutation of isocitrate dehydrogenase in gliomas [65] and renal cell carcinoma [66], and both DESI and REIMS to characterise canine mammary tumours [67].

In addition to metabolic profiling, AIMS has been extensively used in an effort to improve cancer patient outcomes through enhanced surgical resection, guiding surgeons in the evaluation and removal of diseased tissue. Lu et al. used iEESI‐MS to evaluate lung cancer margins. The phospholipid‐rich *m*/*z* 700–900 range was selected for molecular differentiation of cancerous and healthy tissues, which were then subjected to PLS‐DA analysis to build a differentiation model [68]. iEESI‐MS data from tissue located at the tumour margins were then input into the model to determine the disease state of the tissue. Typically, tumour margins are assessed either by subjective visual assessment or by histological examination, the latter of which is a longer process involving freezing, staining and visual interpretation of samples under the microscope. Although only a proof‐of‐concept, this study showed improved tumour tissue detection when compared to a typical histological method, suggesting this method could be used to provide more accurate identification of lung cancer tumour margins to potentially decrease the risk of cancer recurrence. While iEESI proved a potential tool in differentiating healthy tissue and tumours, the vast majority of work in this area in the past has utilised REIMS, a tool primarily developed for use in intraoperative surgery. Vaysse et al. used REIMS for the real‐time intraoperative lipid profiling of soft tissue sarcomas [69]. They took a similar approach comparing metabolite profiles of healthy and diseased tissues using multivariate analysis, but this study differed in the inclusion of in vivo lipid profiling through the analysis of electrosurgical vapours formed during surgical incision. REIMS was furthermore used by Kaufmann et al. for margin assessment of breast cancer, developing classification models with accuracies of 97.1% and 98.6% using different cross‐validation methods [70]. This work performed validation to a higher standard than most equivalent studies, performing analyses across multiple sites in Europe and Canada to demonstrate the robustness of their method. As more studies demonstrate the potential for REIMS in differentiating tissues in real‐time, ongoing efforts are advancing the incorporation of this innovative technology into existing surgical workflows. A recent study demonstrated the first use of REIMS for tissue identification using a surgical robot, with a particular focus on tissues encountered in ear, nose and throat surgeries [71]. The study achieved differentiation of tissue types, including fat, muscle and blood vessels, based on lipid profiles obtained by REIMS whilst using the DaVinci Si surgical robotic platform for accurate, continuous tissue resection. The study demonstrates the exciting potential of achieving the real‐time chemical profiling of tissue in a highly controlled and automated fashion.

**FIGURE 2:**
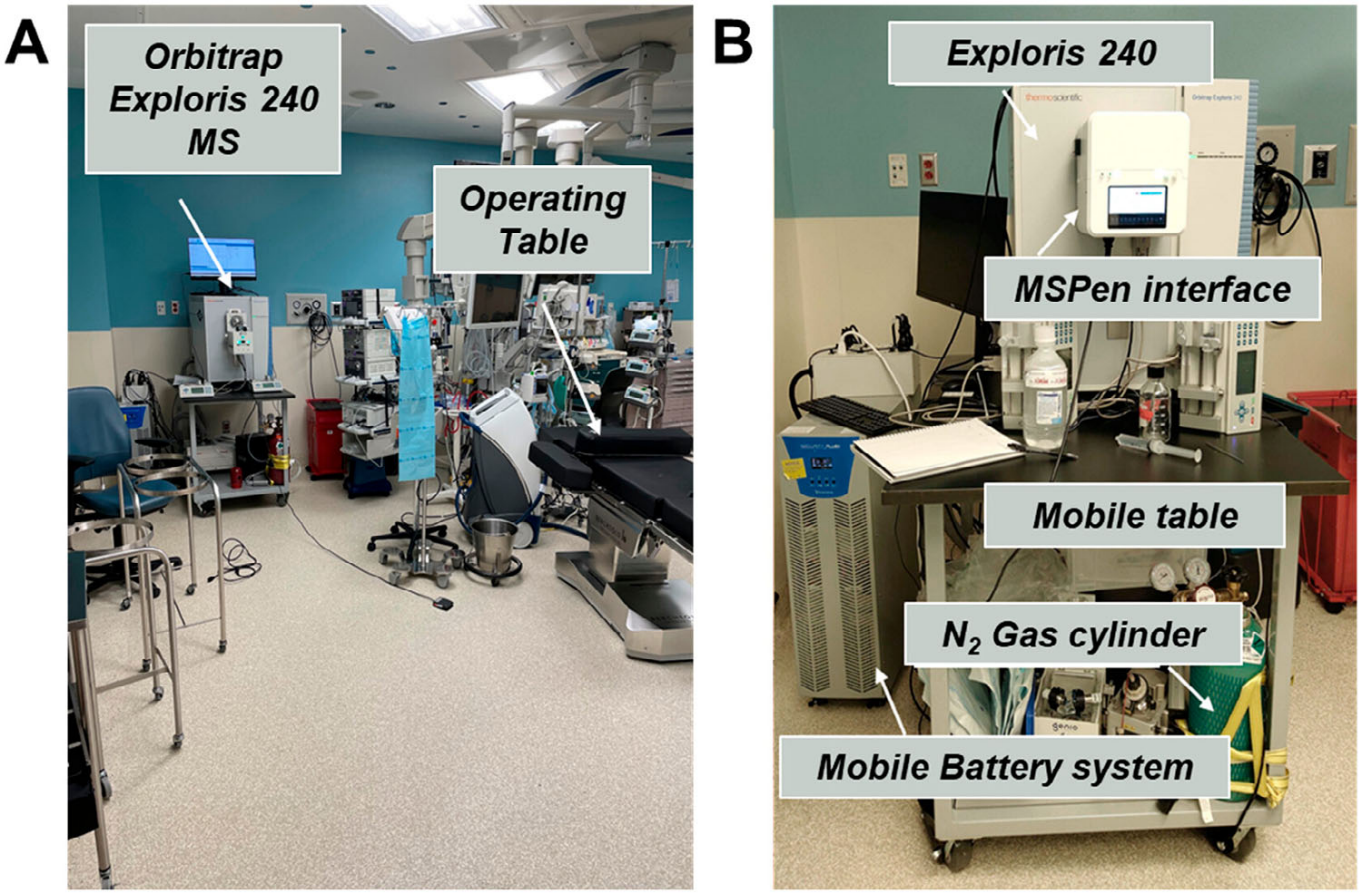
Photographs of a battery‐powered Orbitrap mass spectrometer with a MasSpec Pen interface installed in the operating room at a Texas hospital (A) and the MasSpec Pen interface for interoperative sampling (B). Reprinted with permission from Keating et al. [72]. Copyright 2024, American Chemical Society.

In addition to REIMS, the MasSpec Pen has continued to gain popularity as a tool for improving intraoperative surgeries, which is particularly driven by the Eberlin group. In a recent study, the group discussed the limitations of current intraoperative mass spectrometry instrumentation, in particular, the challenge of installing permanent mass spectrometers in the operating theatre. To overcome this and expand the feasibility of mass spectrometry in the operating room, they coupled the MasSpec Pen with an Orbitrap mass spectrometer operated by battery power for use during ovarian cancer surgery (Figure 2) [72]. This ambitious endeavour enabled the instrumentation to become a mobile system on a wheeled table, which could be transported in and out of the operating theatre and around the hospital. While the study was used to characterise ovarian cancer tissue using the battery‐operated setup, the major focus of the paper was evaluating the effects of instrument mobility on performance. The authors investigated changes in the signal stability and accuracy when the mobile battery‐powered system was moved around the hospital compared to remaining stationary. The TIC signal did not change substantially during movement, with a %RSD of 1.95% when stationary and 4.41% when moved, showing the robustness of the system to maintain stable analytical performance whilst transported around the hospital as a portable system. Other studies have expanded the use of the MasSpec Pen in different areas of cancer research, including Garza et al., who recently used the technique in the analysis of healthy and cancerous breast cancer tissues, with MS‐based conclusions in agreement with postoperative pathology results 96% of the time [73].

The spatial visualisation of analytes in brain tissue can be beneficial in providing valuable insight into neurological conditions, with DESI amongst the techniques most widely reported for this purpose. Of particular interest currently is the use of omics science in developing new diagnostics for Alzheimer's disease. In a recent study by Lv et al., DESI imaging was used to visualise the metabolic changes in areas of the brain where amyloid β accumulation occurs [74]. Amyloid β aggregation is a key process in Alzheimer's disease, thus understanding this mechanism and monitoring its progress is of significant interest for early diagnostics and treatment development. The authors introduce a cold spray DESI method dubbed segmented temperature‐controlled DESI, proposing that sub‐zero temperatures produce a more concentrated spray plume around the target surface, improving the efficiency of desorption and resolution. While the decrease in desorption temperature should logically result in a decrease in sensitivity, in this study, the sensitivity was increased, with the highest signals observed between −10.3°C and −18.2°C in comparison to room temperature. The method was applied to study the molecular changes associated with individual amyloid β aggregates, showing for the first time the depletion of carnosine and increase in 5‐caffeoylquinic acid around the amyloid deposits. Song et al. also used DESI for the visual mapping of metabolites in the brain in an effort to improve understanding of the pathology of depression. Imaging revealed significant changes in the concentration of several metabolites throughout the brain in depressed mice compared to healthy control mice, in particular metabolites related to energy and purine metabolism. Of particular note was deoxyguanosine, which exhibited a differential increase or decrease across different regions of interest in the brain, indicating the potential role of this analyte in the development of depression. Consequently, this approach can enhance our understanding of the impact of depression on the metabolome and support the development of new anti‐depressant drugs. Another fascinating application of DESI for brain imaging is in the study of traumatic brain injury. Leontyev et al. coupled DESI with cyclic ion mobility for the improved separation of isobaric and isomeric species and the reduction of background noise [76]. The combined technology was used to image the brains of rats subjected to traumatic brain injury, with a particular focus on the measurement of small metabolites and lipids. The results of the study showed metabolic changes associated with mitochondrial dysfunction, such as an increase in carnitine in the injured area of the brain. The authors concluded that energy metabolism in the brain may be disrupted by traumatic brain injury, an important finding for understanding brain injury and developing therapeutic treatments. Other recent DESI studies in brain metabolite research have included the detection of biomarkers associated with neurotoxicity using AFADESI [77] and the study of lipidomic changes in the brains of patients with epilepsy by DESI [78].

The capability of ambient ionisation to provide rapid diagnostic information holds the potential to fast‐track the development of treatment plans for patients and improve patient outcomes. Qu et al. developed a rapid screening method for the detection of biomarkers of infertility in urine using desorption, separation and ionisation mass spectrometry (DSI‐MS) [79]. The method did incorporate a brief pretreatment step for the derivatisation of carbonyl compounds; however, this was performed within 1 min on the heated platform of the DSI source, enabling improved ionisation whilst not significantly impacting the rapid analysis time provided by DSI. Across cohorts of healthy participants and patients with various gynaecological diseases, they developed a machine learning model able to not only differentiate healthy from diseased samples but also to detect subtle variations across different gynaecological diseases. The model did, however, struggle to distinguish patients with recurrent implantation failure and recurrent pregnancy loss, likely due to shared reproductive immune system disorders, necessitating further investigation. The same group also used DSI‐MS for the rapid detection of biomarkers of COVID‐19 [80]. They applied the same methodology for the metabolic analysis of urine samples, with a gradient‐boosting machine learning model to classify COVID‐19 patients and healthy control patients that achieved a predictive accuracy of 91%. In this work, they particularly highlighted the compact nature of the DSI source, which could be readily coupled with portable instrumentation for on‐site analysis, though the performance of the ion source with a portable mass spectrometer has yet to be evaluated.

Finally, there are concerted efforts to develop less invasive sampling methods for disease diagnostics to detect disease earlier than traditional diagnostics whilst improving the comfort of patients during testing. The use of exhaled breath has been a particular focus of these efforts, traditionally using GC/MS but, in more recent years, direct analysis techniques such as secondary electrospray ionisation mass spectrometry (SESI‐MS). In a recent study, SESI‐MS was used to characterise the breath profile of lung cancer patients before and after surgery [81]. Over 3000 features were detected across the cohort, with 515 features showing a significant difference pre‐ and post‐surgery. However, after considering the false positive rate, likely to be due to the low sample size, only 154 of these features were determined to be true changes. Perhaps the most significant finding of this study was through principal component analysis, showing a cluster of the profiles of patients who had recurrent or secondary lung cancer, which had been previously undetected by traditional diagnostic and surgical procedures. This finding highlights the exciting potential of MS‐based diagnostics to detect disease in cases where traditional diagnostics fail. Awchi et al. similarly used SESI‐MS for breath analysis, in this case, to study the breath profile of diabetic patients undergoing treatment for diabetic ketoacidosis with the goal of understanding how metabolic pathways are affected during treatment [82]. Known biomarkers of diabetic ketoacidosis were readily detected in the breath samples, highlighting the utility of SESI to detect and monitor the condition. More broadly, metabolic variation before and after treatment was indicative of a shift from a catabolic to an anabolic state, suggesting the effective treatment of ketoacidosis. Although a small pilot study, this work suggests the potential for SESI‐MS to be used in clinical chemistry for both diagnostics and monitoring the effectiveness of the treatment. Finally, in efforts to tackle the challenges in comparing exhaled breath studies brought on by method variation, patient comorbidities, and natural variation, Sasiene et al. set out to establish a typical healthy human breath profile using SESI‐MS [83]. They particularly focused on breath analytes that were variable throughout the day and unique to individuals, which can pose challenges when attempting to identify biomarkers for clinical practice. They furthermore demonstrated age‐related changes in the breath profile, indicating potential for SESI in ageing research.

### 3.4 Biological Materials

Biological materials, such as body fluids and tissues, present significant challenges for mass spectrometric analysis due to their complex composition. Conventional methods such as LC and GC/MS often necessitate lengthy and labour‐intensive sample preparation steps to isolate target analytes, increasing the time and cost of analysis and introducing increased potential for error and contamination. AIMS has gained significant traction in the analysis of biological materials, enabling real‐time, high‐throughput testing without the need for sample pretreatment.

Since its inception, nano‐DESI has been used extensively for the profiling and imaging of tissues, driven by its applicability to biologically complex surfaces and versatility in achieving on‐sample chemical reactions. A key challenge in lipidomics is the structural elucidation of isomers, in particular, the determination of double bond and functional group positions. This challenge is exacerbated in ambient ionisation, which cannot benefit from chromatographic separation. Guo et al. developed a nano‐DESI‐MS [3] method for imaging phospholipid fatty acyl *sn*‐positions and C═C location isomers in animal tissues [84]. Performing on‐tissue photochemical derivatisation followed by analysis with a discontinuous atmospheric pressure injection interface dual‐linear ion trap, they were able to identify over 40 isomers of phosphatidylcholines, phosphatidylethanolamines, and phosphatidylserines, furthermore applying the technique to lipid imaging in diseased liver tissue. In similar efforts to improve isomer and isobar identification in tissue lipidomics, Weigand et al. applied nano‐DESI with a triple quadrupole mass spectrometer for multiple reaction monitoring of lipids in mouse and rat tissues [85]. They achieved the efficient separation of closely spaced phospholipid isobars and eicosanoid isomers in brain and kidney tissues, the latter of which being a poorly studied class of signalling molecules. The same group also used nano‐DESI to target the detection of N‐linked glucans in biological tissues, spatially mapping the challenging analytes for the first time [86]. On‐tissue enzymatic digestion of glycans in human prostate cancer and hepatocellular carcinoma was performed, followed by nano‐DESI imaging of the tissue to detect 38 N‐linked glycans. The authors contrasted the results with those achieved by MALDI, a more commonly utilised technique for tissue imaging, demonstrating a high degree of similarity across the techniques. Other studies have seen nano‐DESI used in combination with infrared photoactivation as an alternative means to decluster protein complexes [87], with a lithium‐doped spray solvent for enhanced ionisation efficiency and analyte identification [88], and applied with the mimetic tissue model, which incorporates analytes into the tissue at known concentrations to account for signal suppression [89].

DESI is used just as broadly in tissue imaging, with recent studies including the enhanced spatial resolution of tryptic peptides in murine tissues [90], imaging the diet‐induced metabolite changes in the kidneys of mice [91], and in a multimodal imaging approach incorporating DESI, SIMS and MALDI [92]. While DESI and its derivatives dominate tissue imaging, other AIMS techniques have also demonstrated utility in imaging. Zhou et al. used a liquid extraction‐based sampling probe to target lipids typically poorly ionisable by traditional imaging techniques due to ion suppression effects caused by matrix analytes [93]. They used a TiO_2_ sampling capillary for the online photocatalytic degradation of interfering phosphatidylcholines. The method achieved the selective photodegradation of phospholipids, preserving other poorly ionisable lipids whose signals would typically be dampened. Finally, Dolatmoradi et al. took a different approach to tissue imaging, opting to image hard tissues, including teeth and hair [94]. Exploring different laser‐based approaches, including LAESI and LDI, they first examined the teeth of rats exposed to tobacco smoke, monitoring the distribution and abundance of nicotine and its metabolites, observing a clear trend in smoke exposure and metabolite levels. In humans, they profiled hair from smokers and non‐smokers, measuring the presence of the nicotine metabolite cotinine in addition to various other endogenous analytes using just single hair strands.

Given the ability of AIMS to detect targeted analytes in a complex matrix, there has been notable interest in the detection of pharmaceuticals in biological matrices, particularly for the purpose of therapeutic drug monitoring, drug discovery and studying drug distribution post‐administration. AIMS has particularly enabled the development of extremely rapid analytical techniques for this purpose. The Cooks group developed two methods for the high‐throughput assessment of bile salt export pump (BSEP) activity, with the goal of developing methods to support drug discovery [95]. They evaluated both DESI and RapidFire‐MS, which incorporates an online SPE process in place of chromatography, isolating BSEP vesicles to evaluate the effects of different drug candidates on BSEP inhibition. In this study, they validated the new methods against LC–MS/MS and radioassays, demonstrating the potential for the two techniques as significantly faster alternatives. The benchmarking of AIMS against standardised methods is becoming an increasingly common practice in analytical method development and a critical step in guiding novel AIMS techniques to more widespread use. In continued drug discovery efforts, the same group has also used AIMS to study opioid binding, developing a label‐free, high‐throughput DESI assay to screen receptor binding of novel opioid analogues [96]. In another study, the Figueiredo group previously developed magnetic particle spray mass spectrometry, applying it to the detection of various beta‐blockers in human plasma [97]. Through the detection of six target analytes, they were able to achieve limits of detection in the low µg/L range at a rate of over 50 samples per hour. Finally, DESI imaging has been shown to be a powerful tool for studying the distribution of drugs in tissues. Three separate studies applied the technique to monitor radiotracer distributions in the brain [98], measure metabolites following the administration of Chinese herbal medicines [99], and study the delivery of the anti‐tumour drug temozolomide to tissue, all of which were performed in rat brains [100]. Similarly, AFADESI has been extensively used to image the spatial distribution of various drugs in tissues, including the anti‐tumour drug nitidine chloride [101], herbal medicines used to treat Alzheimer's disease [102], and the plant‐derived pterostilbene [103], all studied in various rodent tissues.

**FIGURE 3:**
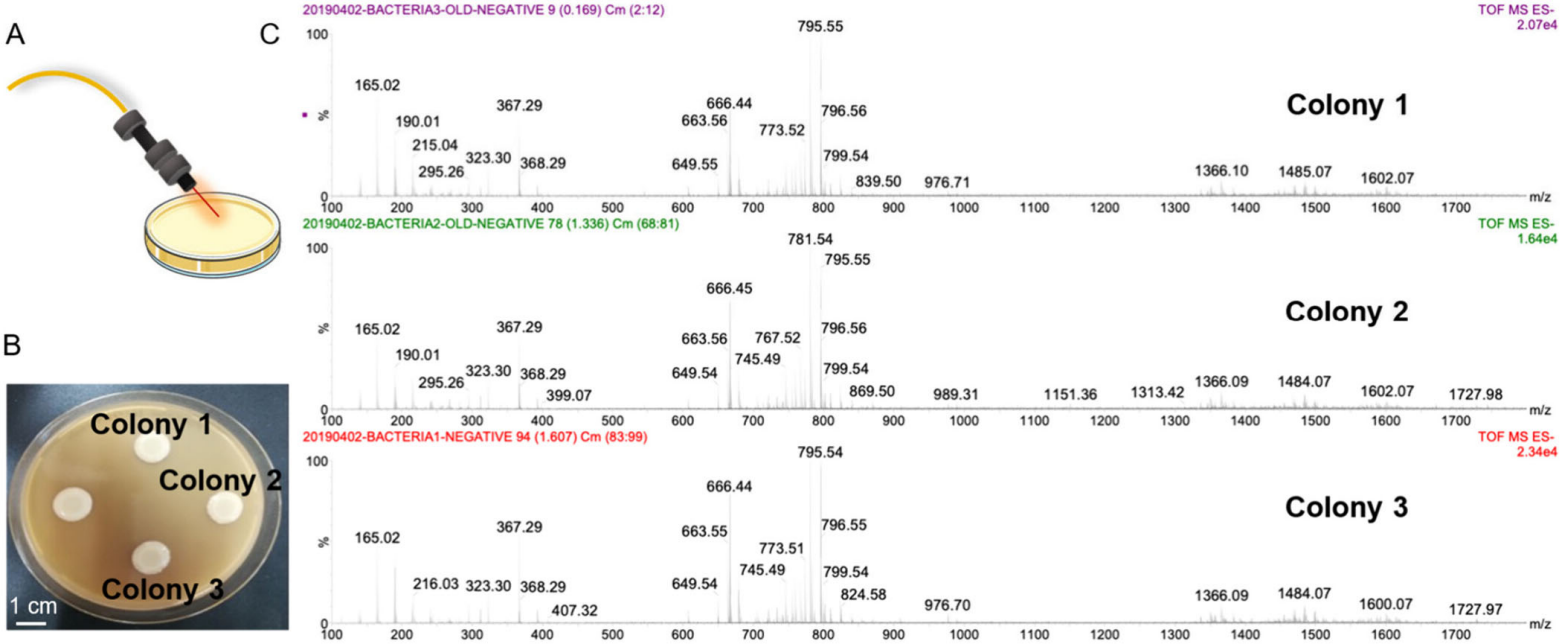
Direct application of SpiderMass to cultures of *Paraburkholderia phytofirmans*. Reprinted with permission from Ogrinc et al. [110]. Copyright 2024, American Chemical Society.

Other non‐human applications of AIMS are becoming increasingly common. While exhaled breath studies have been extensively performed in humans, recent work used SESI‐MS as a non‐invasive method to study rumen fermentation in cows [104]. Monitoring fermentation in the stomach of cattle is important in evaluating livestock health and ensuring nutrient efficiency, but traditional sampling techniques are highly invasive and uncomfortable for the animals. Exhaled breath samples were collected from cattle receiving low and high‐starch diets into Tedlar bags and transported to the laboratory for analysis by SESI. Although there were no striking variations observed between the two diets, the study demonstrated this to be a viable technique for monitoring the exhaled breath of cattle, which could have utility in a range of nutrition and disease applications. In another study, a DART method was developed for monitoring antiparasitics in cattle via the direct analysis of hair [105]. Antiparasitics are frequently administered to cattle to control parasites such as ticks, though the rapid estimation of therapeutic doses can be challenging. Analysis was performed both directly using hair samples by DART and ESI analysis of hair solvent extracts for comparison purposes. Detection of key antiparasitics (fenthion, chlorpyrifos and cypermethrin) was achieved via direct analysis using DART, with no significant matrix effects and suitable limits of detection. Interestingly, DART provided a stronger and more consistent signal in comparison to ESI, a trend not typically observed when comparing ambient ionisation with more traditional, robust techniques. AIMS has furthermore been applied to insects. Tamannaa et al. used DESI to image the distribution of nicotinamide mononucleotide in the Turkestan cockroach *Shelfordella lateralis*, a labile analyte previously challenging to image in tissues [106]. They conducted an initial LC/MS screen of several insect species before further imaging by DESI, performing whole‐body imaging to visualise the localisation of the target analyte to the insect's midgut. The application of AIMS to the study of plants and fungi has also gained increased traction. DESI has been at the forefront of ambient ionisation in such research, recently being applied to map the spatial distribution of amino acids, phenylpropanoids and flavonoids in *Cyclocarya paliurus* leaves [107] and to image metabolites in different regions of the *Cordyceps cicadae* fungus, focusing on the differential distribution of different classes of compounds [108]. You et al. used laser ablation DBD to study pesticide translocation in plants, with the aim of improving pesticide dosing by better understanding the mechanisms behind plant uptake [109]. After exposing tomato plants to the common pesticide difenoconazole, they were able to image the distribution of the analyte throughout the specimen at a spatial resolution of 25 µm, showing the movement of the pesticide through the plant's leaves over a 10‐day period. With no sample preparation other than rinsing, the study demonstrates the potential for plant materials to be collected from the source and profiled for pesticide uptake in a matter of minutes. In similar efforts to reduce the use of pesticides and fungicides, Ogrinc et al. developed a method for the real‐time, in vivo profiling of plants to study plant–microbe interactions [110]. Using grapevines and the bacteria *Paraburkholderia phytofirmans* as a model, they used SpiderMass technology to measure plant metabolites before and after bacterisation, particularly focusing on the localisation of key metabolites in response to the bacteria (Figure 3). They observed the accumulation of flavonols and hydroxycinnamic acids in the leaves and analytes with known relevance to plant resistance to pathogens. The handheld configuration of the SpiderMass probe facilitates non‐invasive sampling of living tissue, theoretically enabling in vivo profiling throughout a plant's life. Other plant‐focused studies have seen neutral desorption EESI‐MS used to monitor metabolic changes in orchid leaves in response to different lighting conditions for optimisation of plant growth [111] and laser desorption DBD for imaging metabolites in *Rheum palmatum* plants with the goal of studying the metabolic variation across different types of the same species [112].

Whilst AIMS has great utility in the analysis of tissues, the past year has seen increased use of the technology to study life on a much smaller scale, targeting microbes. In a recent study, laser desorption REIMS was applied to cultures of *Pseudomonas aeruginosa* bacteria isolated from cystic fibrosis lung infections [113]. Aiming to identify biomarkers of oxidative stress, cultures were grown in the presence of different oxidants and metabolic changes in the cultures characterised, demonstrating a new tool to quickly study the stress responses of bacteria directly from culture. Liu et al. developed a high‐throughput screening platform for the analysis of fatty acid‐producing microbes [114]. In this work, they used laser ablation for the direct ablation of cell colonies, using a nano‐ESI emitter positioned perpendicular to the laser in front of the MS inlet to ionise desorbed analytes. With an analysis time of less than 2 s per sampling point, they were able to rapidly characterise triglycerides produced by the strains, furthermore introducing a custom‐developed Python package for the analysis of LAESI data. Finally, the Oleschuk group developed an LMJ‐SSP platform with hyperspectral visualisation to image cultures of the marine bacteria *Pseudoalteromonas*, a species of recent interest due to its production of potentially useful natural products [115]. The study also incorporated a comparison with direct‐infusion ESI, DESI and MALDI, showing that the LMJ‐SSP method is comparable to commercialised ion sources. Although LMJ‐SSP exhibited poorer spatial resolution in comparison to MALDI and DESI, it was ultimately concluded that the resolution achieved by this technique was sufficient for this application.

### 3.5 Environment

The analysis of environmental samples such as soils, water and plants presents a number of challenges, in particular the complex sample matrices that can necessitate extensive manipulation prior to analysis and the need to transport samples from often remote locations to the laboratory for analysis. As such, AIMS has naturally found a place in environmental sciences, providing both the ability to directly analyse complex materials whilst being well‐suited to coupling with portable instrumentation for on‐site analysis.

In a recent study by Rabinovitch et al., DART‐MS was used to identify different types of petroleum oils, often a subject of environmental concern when oil spills occur [116]. After establishing a sampling protocol by which four classes of oil were collected on hydrophobic paper and subject to DART directly, PCA followed by discriminant analysis was used to create a differentiating model. Interestingly, the study also used weathered oil samples with the model in order to evaluate the possibility of classifying samples that had been subjected to natural degradation processes, attempting to mimic samples that might be encountered in real scenarios. The authors were able to classify weathered and unweathered samples, in addition to the differentiation between different oil types. The use of DART in the study, in addition to the use of paper as a sample collection method as opposed to the use of glass bottles, vastly improved the throughput of collection and analysis compared to typical methods. This could have significant benefits in rapidly investigating and detecting an oil spill in an emergency scenario, consequently protecting marine health. A similar classification method has also been used in a study with sandpaper spray‐ionisation mass spectrometry (SPS‐MS) to distinguish between healthy and infected maple tree leaves [117]. The concept of SPS‐MS is similar to PSI but involves the use of sandpaper as a substrate material, enabling an enhanced surface coverage in comparison to the typical paper surface used in PSI. The healthy and infected leaves were grated against the sandpaper before a high voltage was applied in front of the MS inlet, producing distinct chemical profiles of the leaves. The authors compared the SPS‐MS technique to both leaf spray and PSI, finding SPS exhibited greater stability, thus allowing for more efficient identification of diagnostic ions for differentiation between healthy and infected leaves. AIMS has also been applied to plant leaves for imaging purposes, most recently by Lu et al., who used DESI‐MSI to visualise the distribution of the toxic compound carbamazepine in plants [118]. Carbamazepine was seen to be distributed primarily in the leaves, which allowed further investigation into the effects of the toxic compound in the affected region through metabolomic analysis. Such information could lead to more targeted approaches when both detecting and monitoring toxic substances in plants.

**FIGURE 4:**
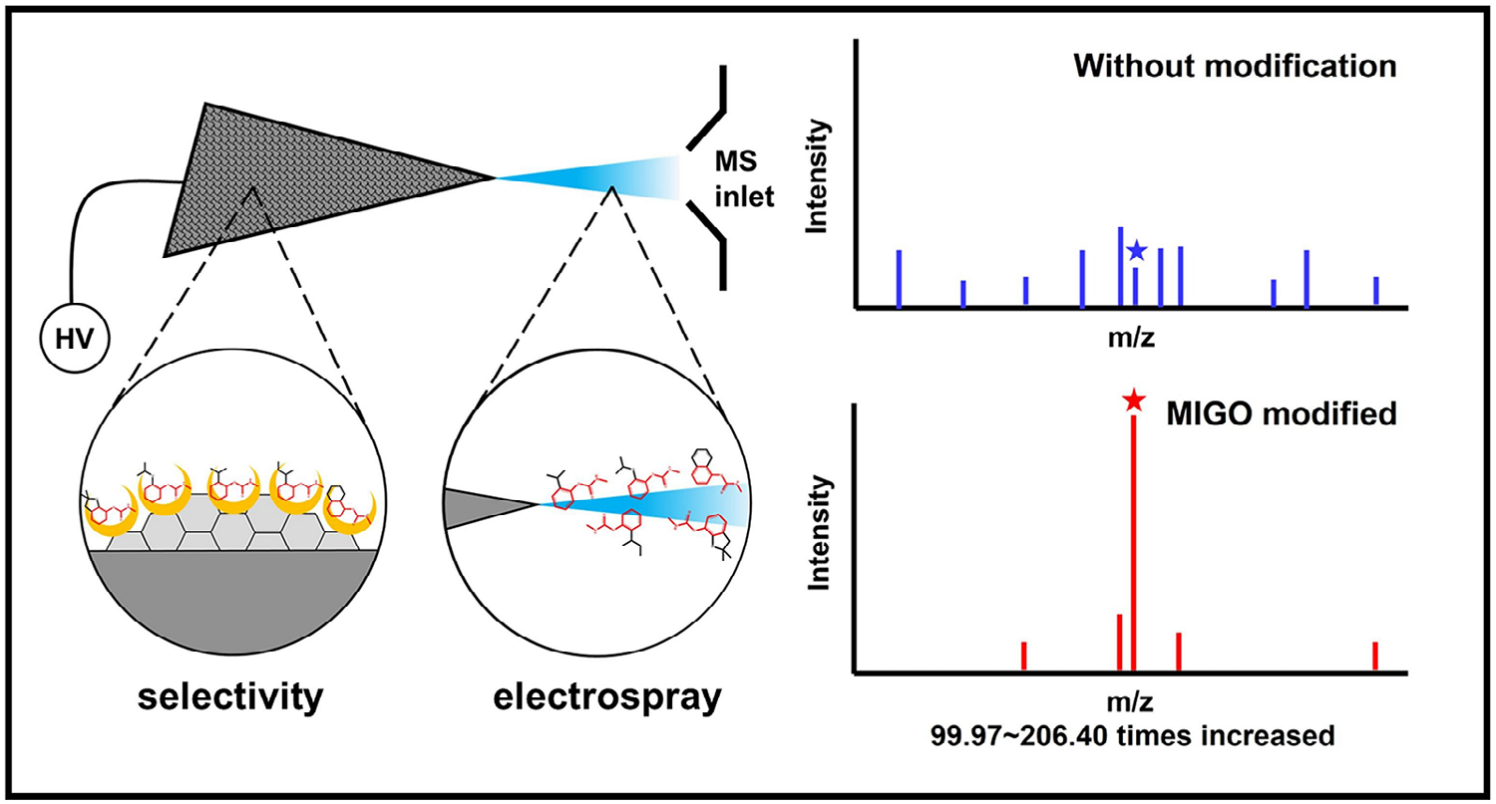
Diagram illustrating the use of molecularly imprinted graphene oxide (MIGO) as a dual‐use ionisation source. Reprinted with permission from Liu et al. [122]. Copyright 2024, Elsevier.

Given the complex nature of environmental samples and the low concentration of target analytes, the development of online sample preparation techniques combined with ion sources has enabled sample cleanup and concentration whilst maintaining the real‐time nature of AIMS. Zhang et al. developed a quantitative method for the detection of antibiotics in seawater using droplet spray ionisation (DSI) [119]. A microsyringe‐based slug‐flow microextraction sample preparation technique (MSFME) was employed prior to analysis, which allowed for a more efficient extraction when compared to traditional liquid–liquid extraction techniques, and the overall analysis time was < 1 min. The authors maintained high sensitivity with their MSFME‐DSI method, with the lowest limits of detection determined to be 0.2 ng/mL. Since the requirement for efficient sample preparation is important when working with complex seawater containing high concentrations of salt, the desalting ability of the MSFME‐DSI method was an imperative part of the study. By comparison of the mass spectra for the antibiotic clarithromycin with and without MSFME (i.e., comparison between MSFME‐DSI and DSI), the results showed minimal signal suppression when pre‐treated using MSPME compared to DSI alone. In a recent publication by Hassan et al. investigating the detection of polyfluoroalkyl substances using desalting paper‐spray mass spectrometry, the throughput of analysis was increased by integrating a desalting step into the ionisation process [120]. This enabled direct analysis of soil samples with enhanced sensitivity, achieving detection of PFAS in the ppt range. Another online microextraction technique utilised in environmental analysis is coated blade spray‐mass spectrometry (CBS‐MS). In a recent study, the authors modified the solid‐phase microextraction coating with novel porphyrin‐based metal–organic frameworks to increase the extraction coverage across polar and non‐polar analytes [121]. In doing so, they were able to quantify 21 drugs of abuse in water samples in the ng/mL range, including opioids, benzodiazepines, and amphetamines. For sample preparation, an automated 96‐well plate was used in this study to maintain the benefits of high‐throughput analysis for the ambient technique. Another study by Liu et al. used molecularly imprinted graphene‐coated stainless steel sheets (MIGOCS) for the selective adsorption of trace carbamate pesticide residues in aqueous environmental samples [122]. The combination of molecular imprinting with ambient ionisation enabled high throughput whilst boosting sensitivity in comparison to unmodified steel surfaces (Figure 4). When compared to other detection methods published for carbamate pesticides, recoveries and LODs were shown to be in line with chromatographic methods (80.2%–97.9% and 0.05–0.4 ng/mL, respectively) but with a greatly reduced total analysis time of 40 s.

The extensive contamination of oceans with hazardous chemicals poses challenges for coral reef ecosystems, and the ability to understand the biological mechanisms of coral reef systems is therefore beneficial to evaluate the impact of chemical exposure. In a study conducted by Iguchi et al., the impact of sunscreen products on coral reefs was investigated by PESI‐MS/MS [123]. The 700 nm diameter needle tip was able to directly sample a single polyp of coral, facilitating sample collection whilst ensuring maximum preservation of the corals in comparison to alternative, more destructive sampling approaches. Coral samples were exposed to different sunscreen products as well as ammonium and nitrates, and the metabolomic profile of the samples was characterised in comparison to unexposed controls to study the metabolic effects of exposure. The authors identified that the model could be scalable to assess the impact of different environmental effects, such as high seawater temperatures, a relevant prospect given the effects of climate change. An additional threat to marine life arises with the increasing exposure of plastics to marine organisms. An iEESI‐MS method was developed to identify any metabolic effects of microplastics on fish exposed to various concentrations of plastics [124]. The authors selected the epidermal mucus as a potential sample matrix due to its rich content, ultimately showing the disruption of 13 metabolic pathways following plastic exposure, in particular those affecting the immune responses and growth. The identification of specific metabolic pathway disruption in this study was beneficial in understanding the impact of chemicals on fish health, and the application of EESI‐MS to the epidermal mucus allowed for a non‐invasive approach with an analysis time of 10 s per sample. Other studies have used AIMS to not only detect plasticisers but also reduce their presence. Gao et al. developed an ambient electric arc ionisation method, which was initially used to investigate plasticisers in food wrap films, items commonly inappropriately discarded in the environment [125]. The technique was first used to quantitatively identify plasticisers in food wrap films with high precision. The authors then used the plasma arc to scan the surface of the food wrap films to decrease the concentration of plasticisers, highlighting the potential dual approach used with the ambient plasma‐based technique by simultaneously detecting and reducing plasticiser content within samples.

Aerosols can have a negative effect on the climate through their ability to affect both solar radiation and impact weather patterns. Organic aerosols are particularly challenging to study. Whilst traditional chromatographic‐based techniques are able to determine the composition of these aerosols, collecting and storing such samples in a way that truly maintains their composition in the atmosphere is impossible. The online analysis of organic aerosols in their original form would provide a more representative profile of their composition. Ge et al. used EESI‐MS to identify the components and potential sources of organic aerosols in eastern China [126]. The EESI technique was used alongside high‐resolution time of flight aerosol mass spectrometry (HR‐ToF‐AMS), to understand whether the ambient EESI approach could provide additional information on the composition of organic aerosols. The analysis from EESI determined the composition of the aerosols to be predominantly C*_x_* H*_y_* O*_z_* and C*_x_* H*_y_* N_1‐2_O*_z_* molecules, with the sources of these molecules including vehicles and biomass burning. When comparing EESI to AMS, it was ultimately determined that the techniques were complementary to one another, with EESI providing a more comprehensive understanding of the formation of organic aerosols but being less suited to the study of hydrocarbons, an important class in organic aerosols. Whilst the identification of the composition of the aerosols is evidently beneficial, there is also the potential to understand the change in the composition of aerosols in real‐time. Kruse et al. used EESI to investigate sea spray aerosols, with a particular focus on the environmental effects on the aerosol properties over time as opposed to their molecular composition alone [127]. The relative humidity over time was found to have a notable effect on the variation of the EESI extraction process, with this effect being associated with the aerosol size and morphology. The EESI signal intensity also varied with changes in relative humidity, which led to the conclusion that this could inhibit the full potential of EESI to identify the molecular composition of the aerosols. The study identified factors, both environmental and aerosol morphological, which could affect the EESI‐MS analysis of aerosols and, therefore, highlighted future work that should be undertaken in this area.

### 3.6 Miscellaneous Applications

Although the vast majority of studies covered in this review fall into forensic science, food chemistry, environmental and biological applications, the versatility of AIMS techniques continues to expand as more fields of research explore the potential of ambient ionisation. Plasma‐based ion sources are amongst the most versatile of the ambient ionisation techniques, being widely applicable to diverse sample types in solid, liquid or gas form. As such, their applications extend far beyond the primary categories of this review. The hazards of cigarettes and, more recently, e‐cigarettes have been a subject of concern for decades, and as such, there has been a notable increase in the use of AIMS to better understand the dangers of their use. Lou et al. used DART‐MS to characterise flavourings in e‐cigarettes, introducing the liquid reservoirs into the ion source via QuickStrip sample cards for rapid sample introduction [128]. They found the sample matrix significantly affected ionisation efficiency, indicating dilution of samples and careful selection of solvents was critical for a quantitative method. The DART‐MS method was furthermore compared to LC/MS, demonstrating comparable performance in terms of quantification, though not all aspects of analytical performance were directly compared. A later study used DART to characterise the composition of cigarette smoke, using a pump‐based system to produce cigarette smoke and SPME probes to concentrate VOCs [129]. They were able to detect over 500 analytes across diverse compound classes and evaluated the chemical variation in inhaled smoke and side‐stream smoke that drifts into the environment to affect others. Other plasma‐based ion sources have also been demonstrated for this purpose, including microwave plasma torch to detect flavour compounds in cigarettes [130]. DBD has also been applied to diverse areas of research. A recent study applied the commercialised DBD‐based SICRIT ion source to the analysis of textiles for the purpose of identification of different materials [131]. They used a soldering iron for the cauterisation of materials, followed by ionisation and analysis, developing a machine‐learning model to differentiate textiles based on their chemical profiles. SICRIT was also used in a recent investigation into the detection of selective androgen receptor modulators (SARMs), which are commonly used for bodybuilding and fitness purposes and prohibited by the World Anti‐Doping Agency [132]. Evaluating the gold standard LC/MS against both SICRIT and DAPPI, the authors found good agreement between the techniques, though they did not perform quantification with the AIMS techniques, rendering them qualitative or semi‐quantitative. They also highlighted that the AIMS techniques would not be suitable for trace analysis given the LODs achieved. Other studies of note include work by the Cooks group, who developed a high‐throughput DESI method to rapidly screen chemical accelerated chemical reactions with the aim of accelerating drug discovery [133]. With 22 starting molecules and over 300 functionalisation reagents, they applied DESI to the analysis of hundreds of reactions occurring under ambient conditions in 384‐well plates. With an analysis rate of 1 reaction per second, they ultimately characterised over 1000 reaction products, once again pushing the boundary of what high‐throughput analysis can achieve. Maiworm et al. used reactive DESI to measure heavy metals in consumer products [134]. The technique was applied to pieces of jewellery, paintings and tableware, using an EDTA solution to form metal–EDTA complexes. Though a novel application of reactive DESI, the technique did not have any significant advantages over the traditionally used x‐ray fluorescence analysis. Surdu et al. applied EESI‐MS to the study of aerosols [135]. Organic aerosols are chemically complex and highly transient, making it challenging to understand their chemistry. By using EESI for real‐time characterisation, the authors were able to measure carboxylic acid adducts to investigate the evolution of aerosols and the effects of their chemical composition on overall aerosol volatility. Finally, Huang et al. used a thermal desorption ESI probe to measure ingested pharmaceuticals via face masks [136]. Face masks worn by participants were used as exhaled breath and exhaled breath condensate collectors following ingestion of acetaminophen. After use, the authors used a handheld sampling probe to collect analytes from the surface of the masks, after which the probe was inserted into the thermal desorption ESI interface for rapid desorption and ionisation. Acetaminophen was detected at its highest 2 h after ingestion before subsequent depletion. The method is shared as a potential means of non‐invasively studying drugs via exhaled breath without the need to collect exhaled breath samples, though it is unclear if this approach would be advantageous.

### 3.7 Technological and Method Advancements

Each year sees the enhancement of ambient ionisation, both through the introduction of novel AIMS techniques and the augmentation of existing techniques to improve reproducibility, robustness and usability. The latter in particular remains a roadblock in transitioning AIMS from the research space into real‐world applications, but each year concerted efforts to enhance technology and methodologies bring us closer to that goal.

Several new ion sources were introduced in 2024, providing novel ways to desorb and ionise analytes in real‐time, often incorporating online sample preparation. Magnetic particle spray mass spectrometry was introduced as a new technique that incorporates online sample preparation into AIMS [137]. MPS uses magnetic sorbent particles to extract analytes from liquid samples, after which they are positioned in front of the MS inlet where a solvent and high voltage are applied to induce an ESI‐like ionisation. In the simultaneous detection of eight common pharmaceuticals in human plasma using deuterated internal standards for improved quantification, low ppb detection limits were achieved with precision typical of an AIMS technique. The integration of online sample preparation with ambient ionisation can reduce ion suppression and improve sensitivity; however, the preparation of the magnetic particles is a time‐consuming multistep process, limiting the throughput of the method as a whole. Chen et al. developed crystallisation and solvent evaporation ionisation mass spectrometry (CSEI‐MS), a technique designed for the rapid desalting of complex samples as part of the desorption/ionisation process [138]. Solid or liquid samples are applied to a heated metal plate to which a nebulised solvent is applied. A rapid crystallisation process occurs, separating analytes from salt crystals and enabling the ionisation of analytes in soil, seawater and hand cream as a result of rapid solvent evaporation. Electroless ionisation (ELI) MS was introduced as an electricity‐free ion source developed for use with portable mass spectrometers [139]. The device simply consists of a syringe and microfabricated self‐ionising nozzle chip, inducing ionisation via electrical charging by the interaction of liquid samples with the nozzle walls. The technique was compared with ESI, achieving comparable results albeit with lower signal intensities. The technique can furthermore be applied to gas phase samples by intersecting the electroless spray with airborne VOCs, inducing an SESI‐like ionisation. One limitation highlighted by the authors was the necessity to use samples with low salt concentrations, with high conductivity samples affecting charge buildup and ionisation efficiency. Desalination of samples prior to ELI‐MS could overcome this issue but would decrease throughput as a result. Future validation of ELI‐MS in applications with samples of high‐salt concentration (e.g., environmental or biological samples) would provide further clarity on these challenges. Tu et al. achieved ionisation by coiling a thin copper wire around the MS inlet with the application of a high voltage, incorporating both corona discharge and electrospray‐like ionisation mechanisms to analyse volatile and semi‐volatile analytes with a range of polarities with sub‐pM concentrations for some analytes [140]. Auvil et al. developed a nanoelectrode APCI source, which incorporates a corona needle 44 times smaller than previously reported and positioned close to the MS inlet for the analysis of a range of gas phase analytes [141]. They demonstrated the potential to achieve both close‐range and non‐proximate sampling of volatiles via tubing ranging from 5 to 15 m. Finally, several new plasma‐based devices have been introduced, including wide‐energy programmable microwave plasma‐ionisation mass spectrometry, which enables the rapid moderation of function waveforms to quickly change ionisation conditions [142], an air‐based low‐pressure alternating current glow discharge ion source for sensitive explosives detection [143], and the development of an inverse LTP source with an ultrasonic nebuliser for sample introduction [144].

In addition to the development of new ion sources, ongoing efforts continue to enhance the original AIMS techniques. PSI is amongst the most widely used AIMS techniques and as such is subject to notable modification and improvements each year. García‐Rojas et al. introduced the Open SprayBot, a custom‐built high‐throughput robotic platform for the automatic analysis of paper‐deposited samples by PSI [145]. The authors developed the system using widely available materials and open‐source technologies, sharing detailed descriptions of its construction and operations to support other labs replicating the system. The system was evaluated through the quantification of palmitoyl‐l‐carnitine, a key biomarker in newborn screening, demonstrating a cost‐effective platform for the rapid screening of drugs or disease biomarkers in biological fluids. Rydberg et al. developed a PSI‐based device that incorporated electrokinetic manipulations on the paper substrate to separate and desalt samples immediately prior to analysis [146]. This approach enabled complex samples to be analysed without sample preparation and with reduced ion suppression effects typically encountered in such matrices, achieving the sub ppt detection of PFAS substances in tap water and < 100 ppt for opioids in urine. Another PSI‐variant termed sorptive tape‐spray ionisation was developed as an alternative approach to performing on‐substrate sample preparation prior to ionisation [147]. This technique uses an aluminium‐based sorptive tape with polymeric microparticles which, upon application of a sample, act as a means to rapidly separate analytes from the sample matrix. The mixed‐mode particles facilitate both ionic exchange and hydrophobic interactions, enabling the analysis of complex samples such as saliva, as demonstrated in this work through the detection of codeine in saliva at sub‐ppb levels. Other PSI modifications have included harnessing the Schiff base reaction for the detection of amino acids in biological samples [148], using plasmonic gold nanoparticle‐treated substrate to enhance analyte detection in a combined PSI‐MS and Raman spectroscopy approach [149], incorporating zinc oxide into paper‐based electrospray devices for enhanced sensitivity [150], developed 3D printed cartridges to enhance usability and reproducibility [151], and used sandpaper to collect and ionise samples [152]. Developments across other AIMS techniques have seen the introduction of a 3D‐printed MS interface and extraction devices for enhancing the usability of CBS [153], the combination of fizzy extraction with SESI for the online extraction of VOCs from liquids [154], modifications to t‐SPESI for enhanced ion transfer and improve spatial resolution [155], and an aptamer‐functionalised magnetic CBS device for the analysis of endoglin [156].

Ion suppression effects have been a longstanding challenge in all ambient ionisation techniques due to the lack of chromatographic separation, particularly in the analysis of samples with a high salt content. In a recent study using LMJ‐SSP in the analysis of bacterial samples, crown ethers were added to the LMJ‐SSP carrier solvent [157]. Crown ethers can bind to metal ions such as Na^+^ and K^+^, both of which are commonly encountered in the AIMS analysis of biological materials. In this work, the LMJ‐SSP probe with crown ether‐doped solvent was applied to the direct analysis of *Pseudoalteromonas* colonies. The addition of crown ethers resulted in a decrease in the complexity of mass spectra with fewer metal ion adducts forming, a twofold to threefold increase in the detection of protonated target analytes, and reduced peak broadening of target analytes, achieving improved resolution of analytes of a similar *m*/*z*. Sun et al. built upon tapping‐mode scanning PESI for mass spectrometry imaging, a technique that uses an oscillating capillary probe to extract analytes through a liquid microjunction and perform electrospray [158]. This most recent study evaluated the effects of oscillation amplitude and phase in the analysis of brain tissue, demonstrating the variation caused by these parameters and their importance in robust imaging. In another study, the efficiency of different gases in DESI was explored, and hydrogen, helium, nitrogen, argon and carbon dioxide were evaluated [159]. It was ultimately determined that hydrogen gave the highest signal intensity, significantly outperforming the more commonly used nitrogen in the ionisation of a range of analytes. The study did however highlight the potential dangers of using flammable gases as nebulisers, a concern which would likely prevent the adoption of these findings. Numerous advancements in the use of SESI for exhaled breath analysis have occurred over the past 12 months. As with many ambient and atmospheric ionisation techniques, quantitative SESI can be challenging and rarely as robust and accurate as traditional MS techniques. To address this, Wüthrich et al. introduced an internal standard addition system for real‐time breath analysis [160]. The modification introduced controlled amounts of gaseous analytical standards into the exhaled breath immediately prior to ionisation and analysis, providing an internal standard for improved quantification of exhaled breath analytes. The same group furthermore explored the use of different electrospray solvents for ionisation of breath VOCs, evaluating dopants based on formic acid, sodium iodide and silver nitrate, showing the silver nitrate dopants enabled detection of completely different metabolite classes compared to the more typically used formic acid [161]. The community is furthermore gradually gaining a better idea of the comparative strengths and weaknesses of different techniques through direct comparison studies. The Sears group has performed thorough comparisons of ASAP, DART, PSI and thermal desorption corona discharge, all coupled with a compact single quadrupole MS [162, 163]. Through the analysis of amino acids, drugs and explosives, they contrasted the respective strengths and weaknesses of each technique, ultimately finding no one technique excelled in all aspects under consideration. Another study previously discussed in the forensics and security section follows a similar trend, comparing different spray‐based techniques (paper and filter cone spray) [46]. Steven et al. performed a comparison of different commercial DESI sources alongside a developmental desorption electro‐flow focusing ionisation source under varying inlet temperatures [164]. In the MS imaging of murine tissue samples, they demonstrated the varying performance of the three sprayer designs in detecting a handful of key analytes in addition to evaluating the usability of each device. The work highlights the variability in different versions of the same technique, adding further complexity to the effective comparison of different AIMS techniques across laboratories. Finally, another recent study analysing tropical fruits compared PSI, direct‐infusion, and tissue spray ionisation, the latter of which uses the sample tissue itself as the spray tip [165]. Targeting the presence of anthocyanins in fruit peel and seeds, they ultimately show that neither ambient technique was as efficient as direct infusion. Combinations of AIMS techniques are also proving effective for tackling poorly ionisation analytes. A recent study incorporated an LTP pretreatment step into a DESI imaging protocol [166]. Brain tissue was first exposed to an LTP source immediately prior to analysis by DESI, with the specific goal of enhancing the ionisation of cholesterol, a lipid key in the study of neurodegenerative disorders. In comparison to untreated tissue, a twofold increase in signal intensity for cholesterol was observed. Whilst not a drastic improvement, the study highlights the interesting potential of combining AIMS techniques to broaden ionisation and detection capabilities.

Given the high cost of many tools and instruments in mass spectrometry and the barrier this creates to widespread adoption, the development of low‐cost ambient ionisation techniques is of particular interest. The Fernández group shared a homemade triboelectric nanogenerator (TENG) ion source, which induces ionisation by conversion of mechanism motion to electric current, demonstrated here for the analysis of lipids and proteins [167]. All materials for the ion source were just over $600 and the authors shared a detailed breakdown of consumables. The technique furthermore boasts relatively low power consumption and a reduced footprint, both key factors when considering the potential portability of technology. The use of triboelectric generators in AIMS continues to diversify, also recently being combined with CBS for trace drug detection [168]. Ion sources produced by laser printers were introduced as a simple and low‐cost ambient ion source [169]. In this technique, a sheet of paper with printed toner spots is positioned in front of the MS inlet, to which a voltage is applied, and volatile and semi‐volatile samples are positioned in between [169]. An electric field is induced between the carbon‐based toner spot and the inlet of the MS, inducing ionisation of volatile and semi‐volatile analytes either positioned near the ion source or applied directly into the toner spot. Gel loading tip spray ionisation was introduced, a low‐cost AIMS device that simply consists of a gel loading pipette tip and a high‐voltage power supply [170]. The pipette tip contains a solvent solution and a platinum wire, the latter of which is used to briefly penetrate a sample to collect a small amount of analyte prior to ionisation by the application of a high voltage. The technique was first applied to the analysis of a number of a series of standards, showing a strong linear response that can often be challenging to achieve with some AIMS techniques. It was then applied to a range of untreated food and pharmaceutical products, demonstrating the ability to rapidly detect a range of analytes with no sample preparation. The authors discuss the new technique in comparison to sfPESI, an extremely similar ion source, though no direct comparison was performed. Finally, Gazeli et al. introduced a low‐cost heat assisted DBD source [171]. The device consists of a simple coil heater and compact plasma sources housed in a 3D‐printed enclosure, in all costs less than €60 and constructable without specialised knowledge in electronics. The study used computational fluid dynamics modelling to explore the impact of heat on gas transport properties critical to analytical performance, ultimately showing the heat‐assist mechanism provided a substantial increase in signal intensity in the analysis of food and pharmaceutical products. They did, however, find the effectiveness of the heat assist was highly sample‐dependent, performing poorly with heat‐resistant samples like olive oil and being affected by samples with high water content.

As the use of IMS technology becomes increasingly available and commonplace, it is unsurprising that researchers are exploring the potential of combining AIMS with IMS. This year saw the first coupling of CBS ionisation with IMS [172]. The CBS ion source enables the online enrichment of target analytes by microextraction and production of ions, after which the ions are drawn into the IMS for separation. The combined techniques were evaluated with mixtures of controlled substances in a mock spiked drink and herbicide in various water samples, enabling rapid separation of target analytes. In coupling the two techniques, the ability of IMS to achieve analyte separation provides that missing feature that is so detrimental to AIMS whilst maintaining the rapid, chromatography‐free nature of ambient ionisation. Another type of IMS, differential mobility spectrometry, has also been coupled with DART‐MS/MS, this study focus on the detection of aromatic amines of concern in various toy products [173]. The combination of DMS with ambient ionisation facilitated the detection of a panel of aromatic amines with reasonable resolution of isomers. Other recent AIMS‐IMS approaches have also seen DESI coupled with cyclic IMS for brain tissue imaging [76].

The vast majority of software for processing mass spectrometry data and identifying unknown metabolites have been designed for traditional chromatography‐coupled techniques such as LC/MS and GC/MS. This poses a challenge for the analysis of datasets produced by real‐time MS such as AIMS, often rendering such programs limited or useless. With this in mind, 2024 has seen an increase in potential solutions to this gap. Wang et al. developed BreathXplorer, a Python package for processing HRMS data produced by real‐time direct analyses [174]. The program is able to detect the start and end points of events (in this case participant exhalation), identify clusters of *m*/*z* values associated with the same metabolic feature, achieve feature alignment across samples, and associate MS/MS spectra with features. This particular study focused on data produced by SESI, but could feasibly be applied to any AIMS technique generating similar data. Similarly, the open‐source Python package Tidy‐Direct‐to‐MS was introduced for handling chromatography‐free MS data [167]. This package incorporates various features, including correction of *m*/*z* drift, feature abundance calculation, and interbatch drift correction. The tool was specifically developed with untargeted metabolomics in mind, though could feasibly be applied to any untargeted AIMS data. The accessibility of python‐based software is however a key limitation of this work, as the expertise required to install and operate the program limits its use to those with knowledge of programming languages. This is particularly restrictive given a key benefit of many AIMS techniques is ease of use by non‐experts. Mansoldo et al. introduced rIDIMS, a browser‐based tool for processing direct‐infusion and AIMS data, offering data alignment, binning, filtering, and grouping of analytes of interest [175]. The tool was particularly developed with usability in mind, intended to facilitate a rapid workflow for users without specialised knowledge of programming.

## 4 Summary and Outlook

With the 20th anniversary of AIMS behind us, the extent of developments in this exciting field of analytical chemistry is evident, and the community has come a long way since the first introduction of DESI and DART in the early 2000s. The ability to perform rapid analysis without sample preparation has broadly revolutionised analytical chemistry. In 2024, alone AIMS has been applied across diverse fields of study, with forensic and biological applications dominating, in particular, biomedical applications for understanding and diagnosing disease.

Subsequent years have seen a marked increase in publications centred around technological and method developments, exhibiting an increase from 12% of total reviewed publications in 2021, to 18% in 2022, and 24% in 2024 [4, 5]. This increase highlights a shift in focus to enhance and develop ambient ionisation techniques through both the introduction of new technologies and software and the improvement of existing techniques. This year has also seen a decrease in publications in the field of food and agriculture from 19% to 9%, whereas biological and forensic applications remain to be the most popular fields in which AIMS is applied. Despite the rapidly changing landscape of ambient ionisation, the AIMS techniques most used remain remarkably consistent. Since 2020, DESI, DART and PSI have been the three most commonly utilised techniques, likely due to their early introduction and commercialisation, though a slight decrease in utilisation of DART was observed this past year.

Several new ion sources were developed including magnetic particle spray ionisation and crystallisation and solvent evaporation ionisation mass spectrometry. Whilst novel AIMS techniques are introduced each year, there has been a notable increase in techniques that incorporate online extraction or cleanup steps, indicating the desire to maintain the rapidity of AIMS whilst achieving minor sample preparation to boost sensitivity and ionisation efficiency. However, the need for specialised materials, such as in magnetic particle spray, can introduce additional preparation steps, slightly offsetting the speed and cost advantages of ambient ionisation. In similar efforts to enhance ionisation, an increased number of studies are incorporating ambient ionisation with IMS, introducing a degree of ion separation or filtration whilst not compromising the real‐time nature of AIMS techniques. CBS‐IMS became the latest technique to be coupled with IMS, improving the specificity of AIMS through enhanced molecular separation [172]. The coupling of AIMS with IMS is becoming increasingly common, particularly with the increased commercialisation of ion mobility instruments. The introduction of ion mobility to AIMS does however add further expense in instrumentation and requires additional expertise in IMS. Aside from enhanced performance, there was further a drive towards decreasing the cost and power consumption of AIMS. Ambient ion sources typically necessitate the use of a high voltage, either to produce an ionising plasma or induce electrospray‐like ionisation. This year saw the introduction of ion sources unique in the absence of a high‐voltage application, with both ELI and CSEI achieving ionisation without additional electronics [138, 139]. The minimisation of high voltage electronics supports both the applicability of ambient ionisation with portable mass analysers for on‐site analysis and the reduction of operational costs.

Finally, this year also saw the introduction of multiple pieces of software geared towards processing data from ambient ionisation, ranging from Python packages to user‐friendly browser‐based programs. The analysis of AIMS data has previously been a challenge, with commercial and open‐source MS software developed for chromatography‐coupled analysis. The increase in data analysis tools highlights the expanding adoption of AIMS and the growing need for more streamlined data‐processing workflows. Aside from new ion sources and software, a large proportion of studies centre around the improvement of existing techniques, through optimised source geometries, chemical modification of spray solvents and source materials, and the combination of complementary technologies. These modifications of existing techniques also looked to address common analytical challenges previously seen with AIMS. For example, the modification of LMJ‐SSP to include crown ethers to reduce ion suppression, a key challenge across all ambient ionisation techniques [157], and the use of electrokinetic stacking in PSI to add an element of cleanup prior to ionisation [146]. Finally, whilst AIMS primarily remains a tool utilised in research, each year sees concerted efforts to optimise its utility in the real world, including implementing portable technologies at festivals [44], evaluating performance with real casework samples [43, 46], and exploring the performance of battery‐operated MS performance for portable use in hospitals [72]. Supported by the increasing commercialisation of ambient ion sources, studies expanding the robustness and performance of AIMS and usability are key in transitioning this technology into the real world.

As the adoption of AIMS expands far beyond what can be covered in this review, 2024 has seen a marked increase in both the general use of ambient ionisation and its enhancement. While there is still extensive progress to be made, each coming year promises exciting growth and development as we look forward to the next twenty years of ambient ionisation.

## Author Contributions

**Alisha Henderson**: writing–original draft (lead). **Liam M. Heaney**: writing–review and editing (equal), conceptualization (equal). **Stephanie Rankin‐Turner**: writing–original draft (supporting), review and editing (equal), conceptualization (equal).

## Conflicts of Interest

The authors declare no conflicts of interest.

## Data Availability

Data sharing not applicable to this article as no datasets were generated or analysed during the current study.
